# Risk Assessment of Cosmetics Using Triclosan on Future Generation’s Germ Cell Maturation via Lactating Mother Rats

**DOI:** 10.3390/ijerph17041143

**Published:** 2020-02-11

**Authors:** Tapas K. Mandal, Nargish Parvin, Sang Woo Joo, Partha Roy

**Affiliations:** 1 Department of Biotechnology, Indian Institute of Technology, Roorkee 247667, India; tps.mndl@gmail.com (T.K.M.); nargish.parvin@gmail.com (N.P.); 2 School of Mechanical Engineering and IT, Yeungnam University, Gyeongsan 38541, Korea

**Keywords:** toxicology, personal care product, male reproduction, androgens, lactation

## Abstract

Triclosan (TCS) is a widely used chemical in personal care and household products as an antimicrobial agent but some studies have reported it as being estrogenic. We investigated the influence of TCS on the male reproductive system of postnatal pups. Lactating mother rats (*Rattus norvegicus*) were given daily doses of 0 mg, 3 mg, and 5 mg/kg/day from the day of delivery until 28 days, equivalent to their natural breastfeeding duration. At 28 days, the male pups of all three groups were sacrificed and their biochemical parameters evaluated. TCS-treated pups had decreased mRNA levels for 3β hydro-hydroxysteroid dehydrogenases (3βHSD), OCT3/4, and androgen receptor (AR) (*p* < 0.05). The higher dose (5 mg/kg/day) male pups exhibited more significantly affected germ cell maturation and decreased body weight. In summary, TCS-treated lactating mothers passed the deleterious effects to their untreated male pups as exhibited by reduced androgens synthesis and subsequently decreased sperm count.

## 1. Introduction

Triclosan (TCS) is used as an antimicrobial additive in personal care and sanitary products such as shampoo, soap, toothpaste, hair gel, floor cleaner, and toys [1,2,3]. The concentration is limited to 0.3% for personal care products [4]. TCS is easily absorbed through the skin [5] and in the gastrointestinal tract following oral ingestion [6,7]. Studies have confirmed the existence of TCS in blood plasma [8], breast milk [8,9], urine [10], and in tissues of the brain, adipose, and liver [11].

TCS may exacerbate estrogenic and/or androgenic effects with an endocrine disruptor. Initial reports of fish showed trends in fin length and sex ratio that weakly suggested androgenic action [12]. In contrast, male fish sperm count decreased and generally modulated female-restricted vitellogenin expression, which is a well-established biomarker of estrogen-dependent and environmental estrogen interaction [13]. It has been reported that prolonged oral intake of TCS (60 days) can reduce sperm production, testes weight, and serum testosterone [14]. In addition, oral TCS doses from 200 to 300 mg/kg can affect testosterone production and male pubertal development [15]. In female rats, oral TCS administration significantly increased the weight of the uterus and stimulated the expression of genes by natural estrogens [16].

On the other hand, a similar experiment showed insufficient growth of the uterine weight of immature females alone with an oral dose of up to 300 mg/kg/day [17]. The period between fertilization and intrauterine implantation of fertilized ova is highly sensitive to fluctuations in natural estrogens. In a careful timeframe, estrogen is in part responsible for preparing both the developing embryo and the uterine environment for implantation [18]. Supraphysiological estrogenic stimulation can affect uterine unification [19], development of the embryo [20], and transport through the oviduct [21]. As evidence, small doses of 17β-estradiol peri-implantation injection can terminate a pregnancy [22].

However, to date, no findings have been reported on the effect on the postnatal germ cell maturation and body growth of TCS when transmitted through breastfeeding. In our experiment, we dosed lactating mother rats with 0 mg, 3 mg, and 5 mg/kg/day of TCS until 28 days after the day of delivery, so that the pups consumed TCS through breastfeeding. The body weight and germ cell maturation of the male pups were significantly influenced by the higher dose (HD, 5 mg/kg/day).

## 2. Materials and Methods

### 2.1. Animals

We used pregnant female Wistar rats (*Rattus norvegicus*) and their male offspring. The procedures used in rearing the rats were approved by the Institutional Animal Ethics Committee (registration number: 563/02/a/CPCSE) and concurred with the UFAW Handbook on the Care and Management of Laboratory Animals. Healthy animals were collected from AIIMS (All India Institute of Medical Sciences, New Delhi, India). They were reared in a temperature-controlled (22–24 °C) animal house in standard 28 cm × 16 cm × 11 cm (height) polypropylene cages with 12 h light: 12 h dark schedule. Sexually mature female rats aged 4–6 months were randomly paired with a male rat of 4–6 months of age. Vaginal sperm plugging was tested three times daily to determine if it had occurred. The sperm-plugging day was denoted as Gestation day 0 (GD 0). On GD 1, each inseminated subject female was housed alone in a clean cage with fresh bedding. Animals were adapted to the animal house condition for 10 days before the experiments.

### 2.2. Dose Selection

**Figure 1:**
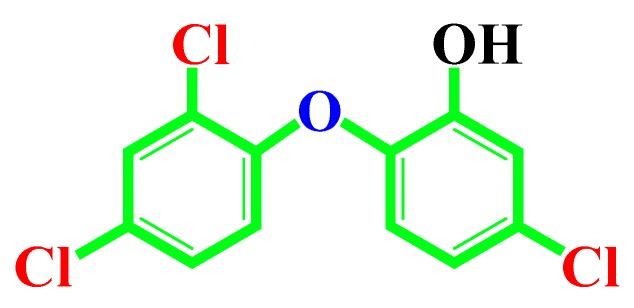
Chemical structure of Triclosan (TCS).

TCS compound (99%) was purchased from SD Fine Chemicals, Mumbai, India (Chemical structure in Figure 1). Selected three doses were taken below LD50 according to previous studies [23]. A homogeneous suspension of TCS in vegetable soybean oil was freshly made every day just before ingestion. Lactating mother rats were given daily doses of 0 mg, 3 mg, and 5 mg/kg/day from the day of delivery until 28 days so that the pups consumed TCS solely through breastfeeding. At 28 days, the male pups of all three groups were sacrificed by cervical dislocation under ether anesthesia. Testes and other accessory sex organs were collected, weighed, and processed according to the experimental requirement.

### 2.3. Treated Chemical Doses

Group I: 0 mg/kg/day (control)

Group II: 3 mg/kg/day (Low Dose-LD)

Group III: 5 mg/kg/day (High Dose-HD)

### 2.4. Daily Sperm Production (DSP)

Testicular sperm content and DSP/g testis were determined from the freshly removed testes of the animals on completion of the treatment according to the method described previously [24].

### 2.5. Immunohistochemistry

Testes were collected from all male pups of the control (TCS untreated) and the two TCS-treated groups and processed further for histopathological analysis. A small spice of testes tissues was set in Bruins solution, dehydrated by upgrading in a series of alcohol from 30 to 100% and finally in xylene each for 1 h. Each dehydrated tissue was then placed in paraffin blocks and cut into sections of 5-µm thickness. Sections were then deparaffinated by incubating twice for 5 min each time in xylene. All methods were described according to the previously published report [25]. The used antibodies’ details are shown in Table 1.

Five-mm-thick tissue sections were placed on glass slides, dewaxed, and rehydrated. Heat-induced antigen recovery was performed in 0.1 M citrate solution (pH 6). Endogenous peroxidase activity was blocked by H_2_O_2_ incubation (3% (*v*/*v*) in methanol for 30 min). Slides were passed into buffer solution (TBS; 0.05 M Tris and 0.85% NaCl, pH 7.6) and finally washed with water. Avidin was used for endogenous biotin blocking, during which a protein block of 5% BSA was incorporated in TBS. This 5% BSA/TBS was used for the dilution of antibodies and applied to the sections at 40 °C overnight in a humidified chamber.

### 2.6. Immunofluorescence Analysis of Testes Tissues

Immunofluorescence was used for staining anti-3βHSD, anti-OCT 3/4, and anti-AR at 1: 1000 dilution. The staining with 3βHSD, OCT 3/4, and AR was visualized by FITC attached goat anti-rabbit secondary antibody. Cells per testis were calculated according to previously reported methods [26].

### 2.7. Image Capture

Fluorescent images were captured using a Carl Zeiss vert.A1 microscope (Carl Zeiss vert.A1 microscope, Carl Zeiss Microimaging GmbH, 07740, Jena, Germany). The intensity of fluorescence was quantified by using the 470nm bandpass (BP) emission filter functions of the Zen imaging software, blue edition (Carl Zeiss Microimaging GmbH, 07740, Jena, Germany). Additionally, the images were organized using Photoshop 7 (Adobe, Mountain View, CA, USA).

### 2.8. RNA Isolation and Semi-Quantitative RT-PCR

Total RNA was extracted from three group experiments (n = 8) by a previously used method [27]. RNA samples obtained from each organism in each group were pooled, quantified, and replicated in equal amounts by UV spectrophotometer OD measurements. Similar patterns of treatment followed by RNA isolation and RT-PCR were conducted three times to minimize internal experimental defects. The temperature for PCR was 94 °C (for 60 s). The annealing temperatures were different due to different primer pairs used. The annealing temperature, primer sequence, and the number of cycles for PCR were determined according to previous reports [28]. The PCR products were isolated using a 2% agarose gel. RT-PCR products were fitted with Scion Image Software (Scion Corporation, Frederick, MD, USA). Each RT-PCR was run three times using β-actin as the internal standard. The primer sequences were designed according to the earlier report [28] and are listed in Table 2.

### 2.9. Statistical Analysis

Results are presented as mean ± S.E.M. The statistical significance was assessed by one-way ANOVA at a 5% level of significance. The statistics software used was Origin 8 (OriginLNab Corporation, Northampton, MA, USA).

## 3. Results

### 3.1. Body and Testis Growth

Treated pups’ body weights were significantly less than that of the control (*p* < 0.05), and this effect was strongest for the higher dose-treated pups (Table 3).

**Figure 2:**
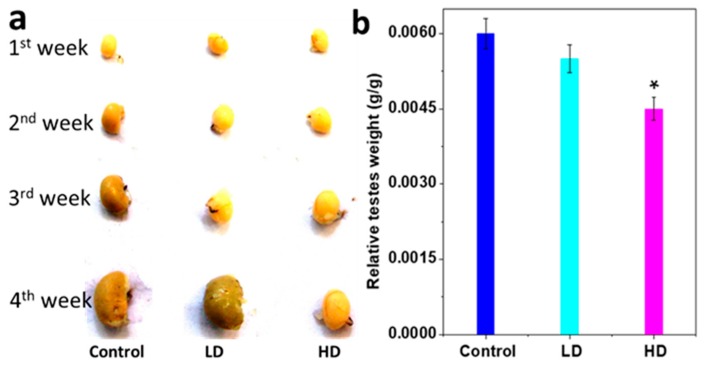
Changes testes response to TCS. (**a**) Representative absolute weekly testes images. (**b**) The histogram results are mean ± S.E.M. of relative testes weight (absolute testes weight/body weight) * significantly different at *p* < 0.05 vs. control.

The testis weight was significantly less for HD than for LD and the control (*p* < 0.05). Relative testes weights (absolute testes weight/body weight, g/g BW) of HD pups were also significantly lower than those of the control and LD (*p* < 0.05) (Figure 2a,b and Table 3).

### 3.2. Immunopositive Staining of Germ Cell Components of Rat Pup Testes

For a better understanding, Table 4 presents the binding nature of the three antibodies (OCT 3/4, 3β-HSD, and AR) with germ cells.

**Figure 3:**
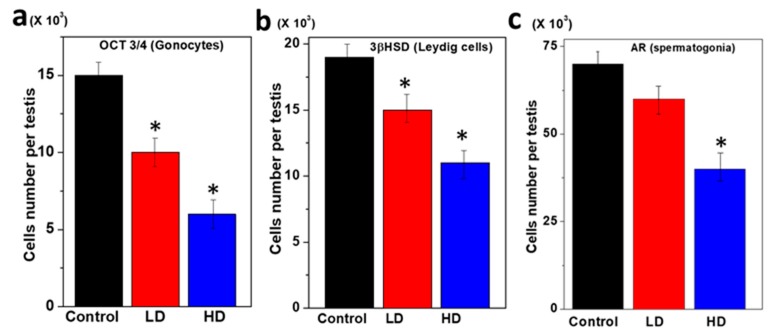
Immunofluorescent staining of germ cells of pup testes after 28 days of breastfeeding. (**a**) Immunopositive gonocytes for OCT 3/4. (**b**) Within the interstitial region of the same set of testes, 3β-HSD-positive Leydig cells and (**c**) AR immunopositive peritubular cell populations.

The germ cell components of the rat pup testes were characterized using specific antibodies and the structural organization compared among the three groups (Figure 3). Small, round single gonocytes were identified as immunopositive for OCT 3/4 in all three pup groups (Figure 3a). The OCT 3/4 was immunolocalized to the nuclei which are the majority of germ cells in the testes. In the testes of the TCS-treated pups, the OCT 3/4 immunopositive staining was more restricted to a subset of germ cells than in the control. Thus, the number of OCT3/4 positive germ cells was significantly reduced (*p* < 0.5) compared to LD and the control (Figure 3a). The surrounding interstitial portion of the testes contained populations of functional Leydig cells positive for 3βhydroxysteroid dehydrogenase [3βHSD] (Table 4).

The staining of the Leydig cells was significantly decreased with increasing TCS dose. The immunofluorescence study for 3βHSD demonstrated that TCS significantly reduced Leydig cell formation activity in the HD dose group (*p* < 0.05) (Figure 3b). Most of the cells (Gonocytes and Spermatogonia) were immunopositive for AR that surrounded the base of the seminiferous cords. Further, the AR-positive germ cell was significantly decreased at HD (5 mg/kg/day) as compared to LD and the control (*p* < 0.05) (Figure 3c).

### 3.3. Daily Sperm Production (DSP)

**Figure 4:**
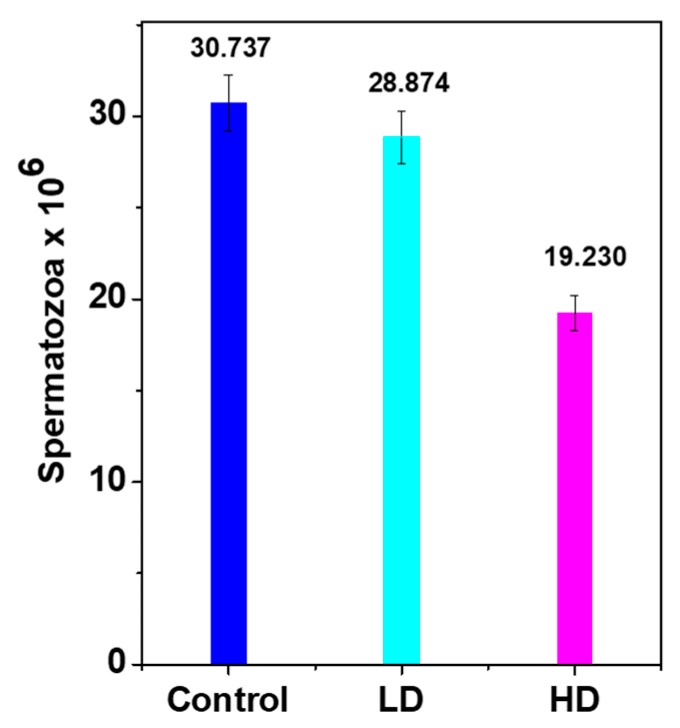
Daily sperm production/g of testis weight (DSP/g) in the control and TCS-treated pups. Results are presented as mean ± S.E.M. (n = 8). The means of DSP were significantly lower (*p* < 0.05) in both treated groups vs. control.

Histopathological findings showed malformations in the HD testes compared with the control, which probably influenced sperm production and maturation. The DSP of both treated groups was lower than that of the control due to low testes weight/growth (Figure 4) that decreased by approximately 6% and 37% for LD and HD, respectively.

### 3.4. Analysis of Gene Expression

**Figure 5:**
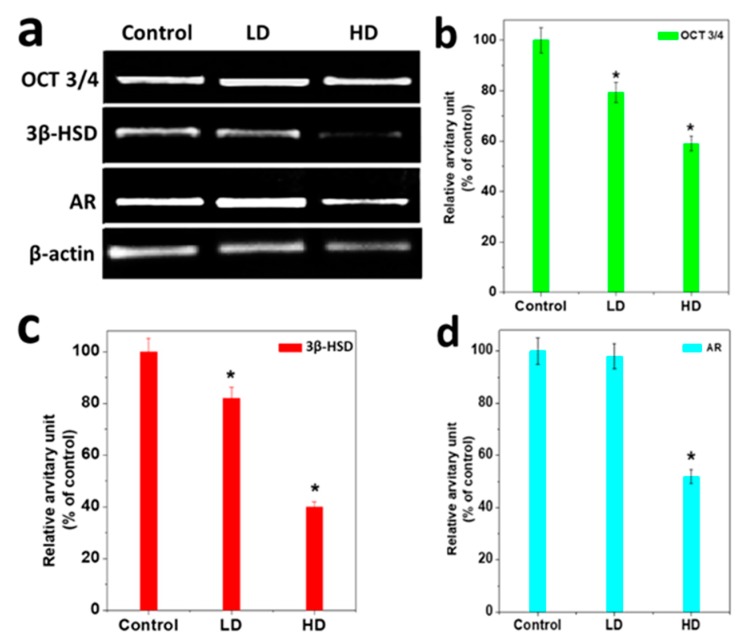
Transcriptional profilesof TCS responses for some testicular genes. (**a**) RT-PCR results of testicular mRNA for OCT 3/4, 3βHSD, and AR genes. All histograms show results as mean ± S.E.M. of the relative arbitrary units of the experimental bands. The control result is denoted as 100%. (**b**), OCT 3/4 genes (**c**), 3βHSD gene and (**d**) androgen receptor (AR). The relative intensities of the signals were quantified by a densitometer and normalized against the internal control (β-actin). * significant results compared to the control at *p* < 0.05.

HD-treated male pups exhibited a down-regulation at the testicular level of mRNA for OCT3/4, 3β HSD, and AR, which differed significantly from the control. OCT 3/4, 3βHSD, and AR mRNA were decreased up to 20% for LD and 55% for HD as compared to the control (*p* < 0.05) (Figure 5a–d). The expression of AR was reduced by up to 46% compared to the control (Figure 5d) (*p* < 0.05).

## 4. Discussion

Despite being a common endocrine-disrupting chemical (EDC) due to the presence of its phenolic moiety, TCS is widely used in commercial cosmetic and plastic manufacturing products as an antimicrobial additive [29,30]. However, the effect on rat pups breastfed by TCS-treated lactating mother rats has not been investigated in terms of germ cell maturation, sperm production, and body growth. In our study, mother rats were orally treated with 0, 3, and 5 mg/kg/day of TCS until 28 days from the day of delivery to examine the effect on the male pups indirectly exposed through breast milk. In the study results, the HD group showed significant responses for all parameters tested, whereas the LD results were almost similar to those of the control.

In the growth study results, HD significantly reduced the weight of the testes and, therefore, may induce testicular hypoplasia and impair spermatogenesis, which is a predictor of infertility or even sterility [31]. The observed body weight was greatly reduced. Similarly, one investigator has reported that EDCs reduce pubertal body weight [32].

The immunopositive staining of germ cells indicated fewer OCT 3/4 positive germ cells at HD than at LD and the control. The 3β hydroxysteroid dehydrogenase [3βHSD] is responsible for functional Leydig cells in the surrounding interstitial portion of the testes. In the results, the Leydig cells staining and AR-positive germ cell staining were significantly reduced at HD compared to the control, which may indicate male infertility. Two previously reported studies have attributed the decline in male fertility to EDC exposure, especially for the chlorinated compounds that are accused of inducing low sperm quality in men [33,34]. EDCs are extracellular agents that interfere with the synthesis, secretion, transport, metabolism, binding and elimination of natural blood hormones present in the body and are responsible for the homeostasis, reproduction, and developmental processes [35]. In this study, DSP results that identified defects in the HD group testes compared to the control were similar to the above reports that demonstrated effects on sperm production and maturation.

Gene expression results revealed reduced transcription and translation of OCT 3/4, 3βHSD, and androgen receptor (AR) mRNA in the testes with increasing TCS dose (as evidenced by RT-PCR and immunofluorescence histochemical analysis). Expression profiles and activity of 3βHSD were also significantly reduced in both treated rat pup groups. This finding is supported by one recent study that showed a direct effect of EDCs at the enzyme level [36].

Several investigators have examined the proliferation of germ cells in rats and human testes. Heimitsch et al. [37] reported that the number of germ cells in the testes increased from approximately 3000 to 30,000, although the proportion of germ cells remained constant in somatic cells. The number of germ cells is associated with mitotic activity in the sperm maturation cycle [38]. Our study has revealed lower expressions of OCT 3/4, 3 β-HSD, and AR-positive cells in the testes of TCS-treated pups, which may have decreased the number of equivalent germ cell signals.

## 5. Conclusions

Previous reports found TCS, a widely used topical bacteriostat, in rat and human breast milk samples. Our study results clearly suggested that TCS in mother rat’s milk acts as a potential endocrine stimulator in the formation of germ cells, such as Leydig cells, Spermatogonia, and gonocytes, in their male pups, as well as affecting daily sperm formation and body growth. Therefore, cosmetics and plastics industries should optimize the concentration of TCS in their products in order to avoid harm to mankind and the environment.

## Figures and Tables

**Table 1:** Details of the antibodies used.

Antigen	Supplier	Species	Retrieval	Dilution
3βHSD	Santa Cruz Biotechnology	Rabbit	No	1:1000
Androgen receptor	Santa Cruz Biotechnology	Rabbit	Yes	1:1000
OCT 3/4	Santa Cruz Biotechnology	Rabbit	Yes	1:1000

**Table 2:** Primers used for semi-quantitative RT-PCR.

Gene	Primer Sequence	Product Size (bp)	Gene Bank Accession No.
3β HSD	F-CCGCAAGTATTCATGACAGA R-CCGCAAGTATCATGACAGA	547	M38178
AR	F-TTACGAAGTGGGCATGATGA R-ATCTTGTCCAGGACTCGGTG	570	M10133
OCT3/4	F:TCACTCACATCGCCAATCAG R:CCTGTAGCCTCATACTCTTCTC	305	M10022
β-Actin	F-TCACCCACACTGTGCCCCATCTACGA R-CAGCGGAACCGCTCATTGCCAATGG	300	NM001101.2

**Table 3:** Comparative study of body and testes weight of the male pups after the completion of 28 days of TCS-treated/TCS-untreated.

Groups	Body Weight (g ± S.E.M)		Testes Weight (g ± S.E.M)
	Initial	Final	
Control (0 mg/kg/day)	7 ± 0.70711	43 ± 0.85391	0.26 ± 0.01813
LD (3 mg/kg/day)	8 ± 0.44721	39 ± 0.91287 *	0.22 ± 0.02225
HD (5 mg/kg/day)	7 ± 0.6	28 ± 1.47196 *	0.13 ± 0.01633 *

Each value denotes mean ± S.E.M. of eight animals. * significantly differ from the control (*p* < 0.05).

**Table 4:** Summary of the binding nature of the specific antibodies with germ cells of male pup testes.

No	Name of Germ Cells	Morphology	Antibodies and Their Nature of Binding		
			OCT 3/4	3βHSD	AR
1	Gonocytes	Small, round, single	positive	Negative	Mostly Negative
2	Leydig cells	Large, elongated, single	Negative	positive	Negative
3	Spermatogonia	Round, single, bids like	Mostly Negative	Negative	Positive
